# Decision Making Profile of Positive and Negative Anticipatory Skin Conductance Responders in an Unlimited-Time Version of the IGT

**DOI:** 10.3389/fpsyg.2019.02237

**Published:** 2019-10-15

**Authors:** Ana Merchán-Clavellino, María P. Salguero-Alcañiz, Fernando Barbosa, Jose R. Alameda-Bailén

**Affiliations:** ^1^Social Psychology Area, Faculty of Education Sciences, University of Cádiz, Cádiz, Spain; ^2^Basic Psychology Area, Faculty of Education Sciences, University of Huelva, Huelva, Spain; ^3^Laboratory of Neuropsychophysiology, Faculty of Psychology and Education Sciences, University of Porto, Porto, Portugal

**Keywords:** decision– making, iowa gambling task, prospect valence learning model, positive anticipatory skin conductance, negative anticipatory skin conductance

## Abstract

Based on the somatic marker hypothesis (Damasio, 1994), many studies have examined whether or not physiological responses are “somatic markers” that implicitly guide the decision making process. Vegetative or motor reactions that are produced by negative or positive stimuli generate a series of somatic markers. So, when a similar stimuli is encountered in the future, these somatic marks will facilitate favorable decisions and inhibit the disadvantageous ones (Martínez-Selva et al., 2006). The most widely studied physiological responses, as indicators of these markers, are heart rate and the skin conductance response (Damasio, 1994; Bechara et al., 1996). The Iowa Gambling Task (IGT) has been the most widely used tool in this research. The common IGT protocol for psychophysiological studies comprises limited inter-trial intervals, and does not distinguish participants as a function of relevant physiological traits, such as the anticipatory skin conductance response (aSCR). The objectives of this work were to determine whether “somatic markers” guide the decision making process without time restrictions and to examine the effects of opposite aSCR profiles on this process. Participants were 29 healthy subjects, divided into two groups according to positive (+) and negative (−) aSCR. Two different data analysis strategies were applied: firstly, gambling indices were computed and, secondly, we examined the parameters of the probabilistic Prospect Valence Learning (PVL) model in three versions: maximum likelihood estimation (MLE), PVL-Delta and PVL-Decay simulations with Hierarchical Bayesian analysis (HBA) for parameter estimation. The results show a significant group effect in gambling indices, with the aSCR+ group presenting lower risk in the decision making process than the aSCR− group. Significant differences were also observed in the Utility parameter of MLE-PVL, with the aSCR− group have low sensitivity to feedback outcomes, than aSRC+ group. However, data from the PVL simulations do not show significant group differences and, in both cases, the utility value denotes low sensitivity to feedback outcomes.

## Introduction

Damasio (1994) attempted to explain through the somatic marker hypothesis why patients with brain damage (ventromedial prefrontal cortex) have poor social functioning, despite achieving adequate scores on other cognitive processes, as assessed by typical neuropsychological tests. This hypothesis argues that decisions are not only determined by rational processes, but also by emotional ones. Thus, a deficit in decision making might be due to difficulties in properly using emotional information from body signals. These body signals, that Damasio called “somatic markers”, would allow regulating or guiding actions toward “good” decisions and they are particularly important in situations of uncertainty, where the exact result of a decision is not known in advance, such as deck selection in the Iowa Gambling Task (IGT).

Vegetative or motor reactions that are produced by negative or positive stimuli generate a series of somatic markers. So, when a similar stimulus is encountered in the future, these somatic marks will facilitate favorable decisions and inhibit the disadvantageous ones (Martínez-Selva et al., 2006). The most widely studied physiological responses, as indicators of these markers, are heart rate and the skin conductance response (Damasio, 1994; Bechara et al., 1996).

The IGT, designed by Bechara et al. (1994), has been consolidated as an assessment instrument of decision making processes under uncertainty. This task consists of four decks of cards (ABCD), with different gains and losses that the subject discovers across the trials. The aim of the “game” is to win as much money as possible, but participants are not informed that there is a hidden strategy. This strategy involves the presence of two advantageous decks (with long-term gains, but each card has a gain or a loss of a smaller magnitude) and two disadvantageous decks (with long-term losses, but each card has a gain or loss of a greater magnitude). The authors propose that decision making can be assessed by computing the Gambling Index (GI), which is calculated by subtracting the choices of the advantageous decks from the disadvantageous ones, that is, GI = (C+D)−(A+B).

The first set of studies based on this task revealed differences between brain-damaged patients and healthy adults, in the sense that healthy people made fewer unfavorable decisions, associated with higher skin conductance in the disadvantageous decks. This was interpreted in the sense that somatic signals intervene in healthy subjects to guide the process of decision making and favor the advantageous decks. On the contrary, higher skin conductance was not observed before choosing the disadvantageous decks in people with brain lesions, indicating the absence of somatic markers that guide the decision making process and leading to worse outcomes on the IGT (Bechara et al., 1996, 1997; Tomb et al., 2002; Carter and Pasqualini, 2004).

In later studies, it has been observed that healthy subjects may also differ in their anticipatory responses, as these may be more or less intense. The “high risk-takers” are defined as having minor anticipatory responses and a poorer performance on the IGT. It is argued that their low physiological responses do not allow the development of appropriate somatic markers (Bechara and Damasio, 2002). However, many questions remain to be solved because various studies report inconclusive results. Differences in physiological responses were not found in all cases: only in individuals with good performance on the IGT (Crone et al., 2004), only in the last moves of the task (Akiyama and Hasegawa, 2014), or only in some decks (Jenkinson et al., 2008). Some researchers rejected the thesis that decision making is guided by somatic markers or emotions (e.g., Maia and McClelland, 2004; Evans et al., 2005), whereas other studies cast doubts on the ecological validity of the IGT (Steingroever et al., 2013). In any case, the inconsistencies in the literature may be due, at least in part, to methodological artifacts.

Concerning the administration time of the task, studies measuring skin conductance responses (SCR) during IGT stipulate inter-trial intervals (ITI) from 7 to 10 s, so finishing the game involves approximately 17 min (Bechara et al., 1996; Bechara and Damasio, 2002; Carter and Pasqualini, 2004; Crone et al., 2004; Jenkinson et al., 2008; Starcke et al., 2009; Fonfría et al., 2015; Ottaviani and Vandone, 2015). However, some studies focused solely on behavioral measures (i.e., no physiological records) in a variety of healthy and clinical samples have not established fixed intervals between the cards, making the IGT a less tedious and more dynamic task (Bechara et al., 1994; Sevy et al., 2007; Buelow and Suhr, 2009; Fridberg et al., 2010; Alameda-Bailén et al., 2014). Considering that both types of studies obtain similar results and are framed within the somatic marker hypothesis, we think that these markers must appear in short temporal intervals controlled by the experimenter, but that it is also important to examine the performance of physiological indices in a more natural process of decision making without time constraints.

In addition, while recording skin conductance provides information about whether emotions play a role in the decision making process, it specifically does not allow us to determine individual sensitivity to the frequency or magnitude of reinforcements and punishments (Bull et al., 2015). Therefore, performance on the IGT can be analyzed by computational probabilistic models, such as (PVL, Ahn et al., 2008, 2011, 2014), complementarily to the Gambling Index. This model, based on Bayesian logic, is based on three general assumptions (Ahn et al., 2008):

– the evaluation of the positive/negative results can be represented by a one-dimensional utility function.
– expectancies about each deck are learned by what is experienced in each trial.
– these expectancies determine the choice probabilities of each deck on each trial.

The PVL model has been applied to different clinical samples and has allowed the identification of distinct decision-making patterns in the IGT (Ahn et al., 2008, 2011, 2014; Alameda-Bailén et al., 2014, 2015, 2017, 2018).

Ultimately, the aim of this study is to investigate whether higher SCR before card selection from disadvantageous decks is associated with better results in the task, and vice versa, with no restrictions concerning maximum response times, as a means to make the decision-making process more natural. In fact, following Bechara et al. (1997), we forced a minimum interval of 1 s before the selection of each card. We also intended to compare the performance patterns of participants with higher and lower SCRs in anticipation of choosing cards from disadvantageous decks using PVL parameters, and to observe possible group differences. In order to better characterize their decision-making styles, we applied the maximum likelihood estimation (MLE), and PVL-Delta and PVL-Decay simulations with Hierarchical Bayesian analysis (HBA) for parameter estimation.

## Materials and Methods

### Participants

Twenty-nine young adults (22 women), aged between 18 and 35 (*M* = 22.31, *SD* = 4.34) participated voluntarily in the study. Participants were recruited among students of the University of Huelva and did not receive any compensation for their participation. We do not keep any personal information of the participants, and we only record their gender and age. This study was carried out in accordance with the Declaration of Helsinki and the recommendations of the Bioethics Committee Guidelines of the University of Huelva, following the protocols established by the university and the Portal of Ethics of Biomedical Research of Andalusia (Portal de Ética de la Investigación Biomédica de Andalucía: PEIBA), although, ethical approval was not required in line with national legislation and institutional guidelines. All subjects gave written informed consent in accordance with the Declaration of Helsinki.

After performing the task, participants were further divided into two groups, according to their anticipatory SCR: (a) the aSCR− group comprised 14 participants (9 women), with an average age of 21.43 (*SD* = 3.65) and a baseline mean SCR of 4.82 μS (*SD* = 3.25); (b) in the aSCR+ group, there were 15 participants (13 women), with an average age of 23.13 (*SD* = 4.88) and a baseline mean SCR of 6.66 μS (*SD* = 4.46). In order to compute individual aSCR values, skin conductance preceding each card (1250 ms time-window) was averaged, and the inverse GI formula was applied (Carter and Pasqualini, 2004), that is, SCR in anticipation of disadvantageous choices (A+B) minus the responses to advantageous choices (C+D): aSCR (A+B) – aSCR (C+D). Results of this formula are either positive or negative, with positive aSCR values representing higher conductance responses in anticipation of disadvantageous decks, and negative values meaning higher responses in anticipation of advantageous decks.

No significant group differences were observed for age, *t*_(27)_ = −1.06, *p* = 0.299, sex, χ^2^ = 1.98, *p* = 0.159, and skin conductance during the period of no stimulation (baseline), *t*_(27)_ = −1.26, *p* = 0.217.

## Task

We used the *Cartas* software (Palacios et al., 2010), a computerized version of Bechara et al. (1994) IGT, “ABCD” version. The task consists of presenting four decks of cards (A, B, C, and D), from which the subject must choose one card in every trial for a total of 100 choices. Each deck has a total of 40 cards displaying a certain gain or loss. The participant starts with the amount of €2000, displayed on the screen, which is updated with the gains or losses after each trial.

For every 10 cards selected from the disadvantageous decks (A and B), the subject loses a total of €250. In deck A, gains are always €100, but losses range between €150 − 350 in 5 out of 10 elections, whereas in deck B, there is a single loss of €1250 for each cycle of 10 cards.

For every 10 cards selected from the advantageous decks (C and D), participants gain €250. Losses in deck C range between €25 – 75, whereas in deck D, there is a single loss of €250, occurring once every 10 cards. The aim for participants is to win as much money as possible, although the best strategy is concealed when giving the instructions.

After selecting one card, participants had to wait at least 1 s before choosing the next one, but they had no time limit for their responses.

### Procedures

Data gathering sessions were performed individually, with an approximate duration of 45 min, in a room with adequate lighting and acoustics. All participants were informed about the objectives of the study and gave informed consent before starting the experiment.

Afterward, the participant was seated comfortably in front of a computer and prepared for physiological recording. Skin conductance (SC) was measured using MC-6SY cable and compatible electrodes (range 5–100 μS) connected to an I-330-C2+ 12-channel polygraph, synchronized with USE3 Physiolab data processing software (all from J & J Engineering, Inc., Washington, DC, United States).

Electrodes were placed on the middle phalanges of the index and middle finger of the non-dominant hand (JE-26 gel was employed). Participants were instructed not to move the hand and to remain silent during the experiment. A baseline acquisition (1 min) was performed before starting the IGT, and the SC recording was run simultaneously with the task thereafter. Markers were manually inserted in the recordings each time participants selected a card.

In order to examine the decision-making processes, measures from two analytical procedures were computed. Firstly, we obtained the GI, as described above, and other classical IGT measures. Partial GIs were also calculated for blocks of 20 trials each, that is: B1 (cards 1–20), B2 (cards 21–40), B3 (cards 41–60), B4 (cards 61–80), and B5 (81–100). Thus, we analyzed the following measures: total GI, partial GIs, number of choices per deck (ABCD) and number of choices per type of deck (advantageous vs. disadvantageous). Secondly, we determined the following PVL parameters: utility, loss aversion, recency, and consistency (see Table 1).

**TABLE 1:** Summary of prospect valence learning model (PVL).

	**Interval of values**				
**Parameter**	**Maximum likelihood**	**Decay Rule**	**Delta Rule**	**Minimum value**	**Maximum value**
Utility (α)	0 < α < 1	0 < α < 2		Sensitivity to feedback outcomes	
				Lower	Higher
			
Loss aversion (λ)	0 < λ < 5	0 < λ < 10		Sensitivity to losses relative to gains	
				Higher	Lower
		0 < A < 1			
Recency (A)	Decay Rule	Decay Rate	Learning Rate	Recent outcomes	Past outcomes
Consistency (c)		0 < c < 5		Random	Deterministic

The equations to calculate the parameters of PVL are:

To rate a card:

(1) $$u\,\left(t\right)=\left\{ \begin{array}{c} x\,\left(t\right)\,x^{a}\to \mathrm{if}\,X\,\left(t\right)\geq 0\\ -\lambda \,{\left|x\,\left(t\right)\right|}^{\alpha }\to \mathrm{if}\,X\,\left(t\right)<0 \end{array}\right.$$

where:

**α = Utility or Reward sensitivity**. This regulates the shape of the utility (power) function. High values of α indicate more sensitive to feedback outcomes, whereas low values of α indicate low sensitivity to feedback outcomes.

****λ** = Loss aversion**. This determines sensitivity to losses compared to gains. A value of λ less than 1 indicates more sensitivity to gains than to losses whereas a value of λ greater than 1 indicates more sensitivity to losses than to gains.

To create deck expectancy, E, for deck *j* on trial *t*, the equation for decay-reinforcement rule is:

(2) $$E_{j}\,\left(t\right)=A\cdot E_{j}\,\left(t-1\right)+\delta_{j}\,\left(t\right)\cdot u\,\left(t\right)$$

and the equation for delta rule is:

(3) $$E_{j}\,\left(t\right)=E_{j}\,\left(t-1\right)+A\,\delta_{j}\,\left(t\right).\left[u\,\left(t\right)-E_{j}\,\left(t-1\right)\right]$$

Where *j* refers to deck A, B, C, or D. δ*j(t)* is a dummy variable equal to 1 if deck j was chosen on trial t, and otherwise is 0. A is the recency or learning rate parameter.

***A* = Recency** **parameter/learning rate.** In PVL-Delta the expected value is updated with a learning rate parameter and a prediction error term. Where *A* close to 1 places more weight on recent outcomes, and where *A* close to 0 places more weight on past outcomes. The difference between predicted and experienced outcomes is the prediction error. In PVL-Decay A is used for value updating. The recency parameter indicates how much the expected values of all decks are discounted on each trial.

In the delta rule (Rescorla–Wagner rule, Rescorla and Wagner, 1972), only the expectancy of the selected deck is updated while the expectancies of other decks remain unchanged. In the decay rule (Erev and Roth, 1998), A is used for value updating. It indicates how much the expected values of all decks are discounted on each trial. The decay rule permits the expectancies of all the alternatives to change on each trial, thus is more flexible than the delta rule but high model flexibility may over-fit the data and lead to poor generalizability.

The equation to calculate the probability of choosing Deck *j* is:

(4) $$Pr\left[D\left(t+1\right)=j\right]=\frac{e^{\theta \,\left(t\right)\cdot e_{j}\,\left(t\right)}}{\sum_{k=1}^{4}e^{\theta \,\left(t\right)\cdot E_{k}\,\left(t\right)}}$$

and, finally, to calculate the consistency between choices and expectancies, the equation is:

(5) $$\theta \,\left(t\right)=3^{c}-1$$

where: ***c* = Consistency or Response Sensitivity**. This is a consistency parameter (choice sensitivity), it reflects how deterministically individual choices are made in relation to alternative choices. High values represent more deterministic choices and low values random choices.

We applied the (MLE, Ahn et al., 2008), and PVL-Delta and PVL-Decay simulations with HBA for parameter estimation (Ahn et al., 2008, 2014, 2016).

The MLE has been performed with the decay rule (Erev and Roth, 1998), as it consistently shows better models of *post hoc* fit than the delta rule in the IGT (Yechiam et al., 2005). For the MLE estimation of PVL parameters we follow a scrip in R programmed by Ahn et al. (2008).

Hierarchical bayesian analysis simulation method uses the parameters estimated from the IGT task as seed to make predictions. It is not an individual level analysis. To perform HBA, we used a R packages hBayesDM (Ahn et al., 2016) and RStan (Stan Development Team, 2014), which uses Markov chain Monte Carlo (MCMC) sampling Hamiltonian Monte Carlo (HMC) algorithms, that allows efficient sampling for complex models and with highly correlated parameters. The individual parameters were obtained from the normal distributions at the group. On the websites:

– https://www.rdocumentation.org/packages/hBayesDM/versions/0.4.0/topics/igt_pvl_delta
– https://www.rdocumentation.org/packages/hBayesDM/versions/0.4.0/topics/igt_pvl_decay

you can see the commands used for both HBA simulations: PVL-Delta and PVL-Decay. The HBA simulations were performed based on of each group data, thus, we performed for the aSCR− group the corresponding HBA simulations (PVL-Delta and PVL-Decay), and equally, the HBA simulation, PVL-Delta and PVL-Decay, for aSCR+ group (using the procedure in Appendix B of Ahn et al., 2008).

Statistical analyses comprised: (a) *t*-test for independent samples to examine possible group differences in decision making (aSCR+ vs. aSCR−) in the total test (GI); (b) repeated measures ANOVA followed by planned contrasts to analyze group differences in partial GIs (B1, B2, B3, B4, B5) to observe task evolution across the blocks; (c) repeated measures ANOVA followed by planned comparisons of groups (aSCR+ vs. aSCR−) to examine differences in the number of choices depending on the deck type (advantageous vs. disadvantageous) and deck (A, B, C, D); (d) Student’s *t*-test for independent samples on PVL parameters; (e) repeated measures ANOVA of SRC activation (anticipatory/post-election) by bloks, for the positive (aSCR+) and negative (aSCR−) anticipatory skin conductance response groups for advantageous and disadvantageous desk; and (f) repeated measures ANOVA of the deck choice times by block for the positive (aSCR+) and negative (aSCR−) anticipatory skin conductance response groups for advantageous and disadvantageous deck (see Supplementary Data Sheet S1).

## Results

**FIGURE 1:**
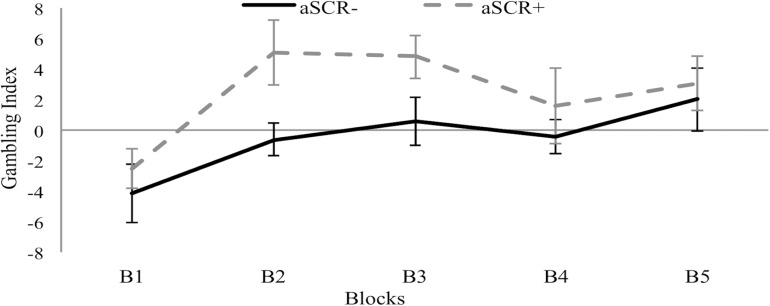
Means of the Gambling Index by block for the positive (aSCR+) and negative (aSCR–) anticipatory skin conductance response groups (error bars represent standard error of the mean).

The results showed significant differences between the aSCR+ group (*M* = 12.00, *SD* = 18.99) and the aSCR− group (*M* = −2.29, *SD* = 13.38) for total GI, *t*_(27)_ = −2.33, *p* = 0.028, *d* = 0.41. Concerning the partial GIs, the results show a group effect, *F*_(1,27)_ = 5.58, *p* = 0.026, η^2^ = 0.17, with the planned contrast analyses revealing significant differences in block B2, *t*_(20.4)_ = −2.42, *p* = 0.025, *d* = 0.47, and a marginally significant difference in B3, *t*_(27)_ = −1.98, *p* = 0.058), *d* = 0.36, with higher partial GI scores for the aSCR+ group in both cases (see Figure 1).

Regarding the effects of group (aSCR+, aSCR−) and deck type (advantageous, disadvantageous) on the number of choices, the repeated measures ANOVA did not show any main effect (*F* < 1) but we found a significant interaction effect of Group^∗^Type of Deck, *F*_(1, 27)_ = 5.41, *p* = 0.028, η2 = 0.17. Specifically, whereas the aSCR+ group chose more cards from the advantageous decks (*M* = 56.00, *SD* = 9.49) than from the disadvantageous ones (*M* = 44.00, *SD* = 9.49, *p* = 0.009) the aCSR- group chose similarly from both types of decks, with a non-significant tendency (*p* > 0.05) to select more cards from the disadvantageous decks (*M* = 51.14, *SD* = 6.69) over the advantageous ones (*M* = 48.86, *SD* = 6.69).

Planned comparisons revealed significant group differences both for the advantageous and disadvantageous decks, *t*_(27)_ = −2.33, *p* = 0.028, *d* = 0.41.

In a further analysis of the effects of group (aSCR+, aSCR−) and deck (A, B, C, D) on the number of choices, the repeated measures ANOVA yielded a main effect of deck, *F*_(3, 81)_ = 25.27, *p* < 0.001, η^2^ = 0.48, and a marginal interaction effect was also obtained, *F*_(3, 81)_ = 2.58, *p* = 0.059, η^2^ = 0.08. *Post hoc* analyses within the aSCR− group revealed significant differences between decks A–B (*p* < 0.01), A–D (*p* < 0.05), and B–C (*p* < 0.05), whereas the aSCR+ Group showed differences between decks A–B (*p* < 0.01), A–C (*p* < 0.01), and A–D (*p* < 0.001).

**FIGURE 2:**
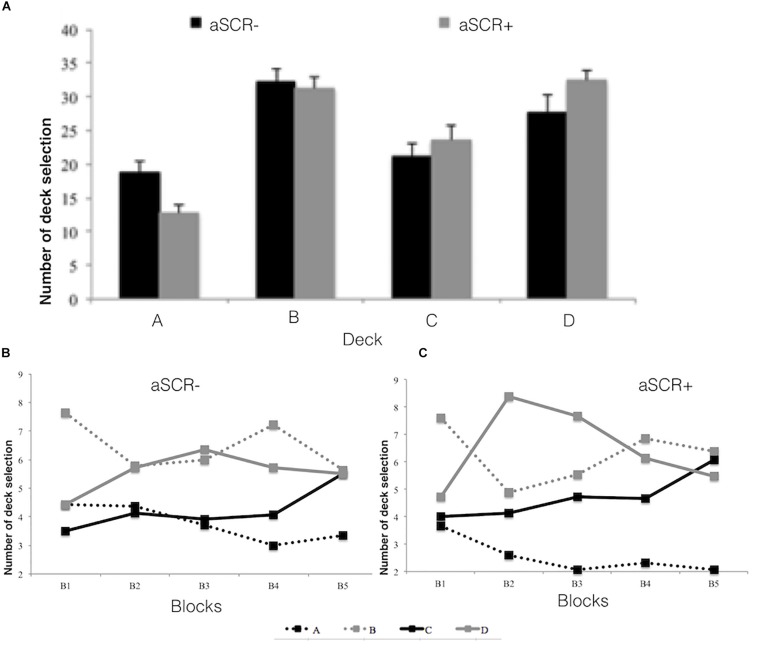
Number of deck selections (ABCD) for the positive (aSCR+) and negative (aSCR–) anticipatory skin conductance response groups (error bars represent standard error of the mean), for total task **(A)** and by blocks **(B)** for (aSCR– and **C** for aSRC+).

Planned comparisons revealed a significant group difference only for deck A, *t*_(27)_ = 3.19, *p* = 0.004, *d* = 0.59, which was more frequently selected by the aCSR- group (see Figure 2).

Regarding to the parameters of the PVL model, as can be seen in Table 2, there were no significant differences between aSCR− and aSCR+, in PVL-decay and PVL-delta simulations, the parameter utility (**α**: *t* = 2.432, *p* = 0.006) presented significant differences using MLE.

**TABLE 2:** Descriptive and statistical analysis of the PVL parameters.

	**MLE:** **PVL**			**HBA: PVL-Decay**			**HBA: PVL-Delta**		
	**aSCR-**	**aSCR+**		**aSCR-**	**aSCR+**		**aSCR-**	**aSCR+**	
	**M (SD)**	**M (SD)**	**Sig.**	**M (SD)**	**M (SD)**	**Sig.**	**M (SD)**	**M (SD)**	**Sig.**
A	0.495 (0.31)	0.458 (0.32)		0.918 (0.05)	0.919 (0.05)		0.093 (0.06)	0.092 (0.06)	
**α**	0.557 (0.41)	0.165 (0.26)	0.006	0.529 (0.26)	0.534 (0.25)		0.680 (0.21)	0.681 (0.21)	
c	0.365 (0.36)	0.998 (1.16)		0.430 (0.98)	0.431 (0.98)		2.008 (0.68)	2.010 (0.68)	
**λ**	3.00 (2.17)	2.57 (2.22)		1.369 (0.53)	1.358 (0.52)		1.001 (0.40)	1.001 (0.39)	

*A (recency); α (utility); c (consistency), and λ (loss aversion).*

**FIGURE 3:**
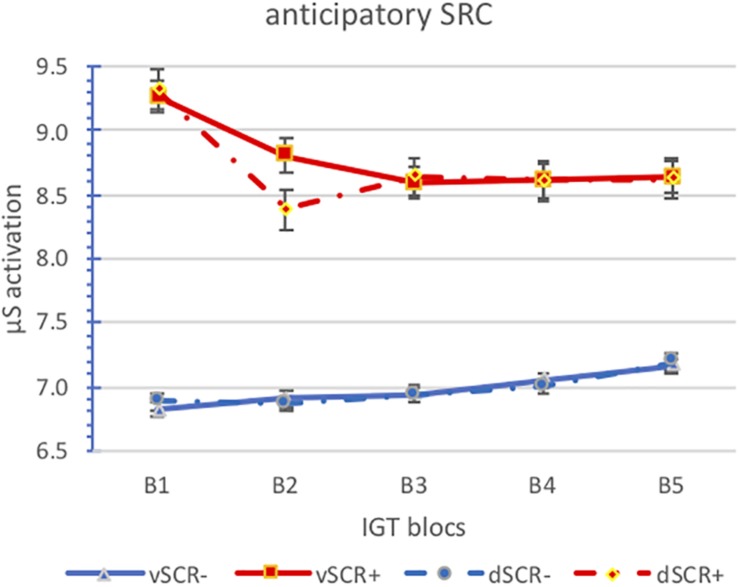
Means of the anticipatory SCR activatión by block for the positive (aSCR+) and negative (aSCR–) anticipatory skin conductance response groups for advantageous and disadvantageous desk (error bars represent standard error of the mean).

**FIGURE 4:**
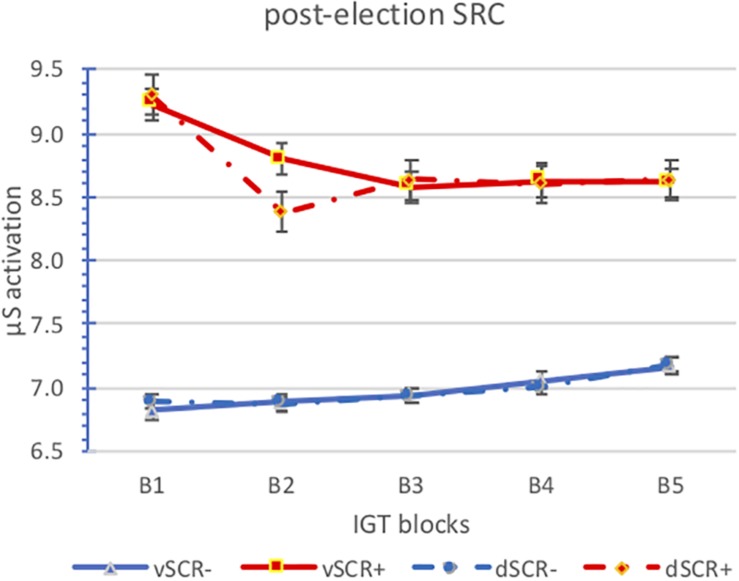
Means of the post-election SCR activation by block for the positive (aSCR+) and negative (aSCR–) anticipatory skin conductance response groups for advantageous and disadvantageous desk (error bars represent standard error of the mean).

We have analyzed the anticipatory and post-election SCR activation by block for the positive (aSCR+) and negative (aSCR−) anticipatory skin conductance response groups for advantageous and disadvantageous desk (Figures 3, 4 and Table 3).

**TABLE 3:** Mean and S.D. of anticipatory and post-election SCR activation for advantageous and disadvantageous decks by blocks.

	**Anticipatory SCR**								**Post-election SCR**							
	**Advantageous (C + D)**				**Disadvantageous (A + B)**				**Advantageous (C + D)**				**Disadvantageous (A + B)**			
	**SCR-**		**SCR+**		**SCR-**		**SCR+**		**SCR-**		**SCR+**		**SCR-**		**SCR+**	
	**Mean**	**S.D.**	**Mean**	**S.D.**	**Mean**	**S.D.**	**Mean**	**S.D.**	**Mean**	**S.D.**	**Mean**	**S.D.**	**Mean**	**S.D.**	**Mean**	**S.D.**
B1	6.821	3.914	9.263	7.629	6.880	3.910	9.314	7.714	6.820	3.904	9.235	7.562	6.888	3.897	9.301	7.684
B2	6.902	3.903	8.806	7.489	6.860	3.896	8.388	7.639	6.887	3.888	8.793	7.511	6.875	3.911	8.382	7.674
B3	6.942	3.926	8.591	7.521	6.930	3.912	8.638	7.476	6.943	3.934	8.581	7.502	6.930	3.905	8.635	7.490
B4	7.047	3.981	8.607	7.742	6.996	3.936	8.611	7.750	7.057	3.979	8.625	7.769	7.005	3.938	8.602	7.758
B5	7.162	4.073	8.634	8.357	7.192	4.105	8.626	8.529	7.174	4.087	8.610	8.319	7.185	4.097	8.637	8.550

We can see how the activation levels of the aSRC+ group are higher than those of the aSRC− group, however, the activation levels between advantageous and disadvantageous choices are similar in both groups. Also note that there are no differences between anticipatory and post-election activation. Perhaps it should be noted that while we can observe a downward trend in the aSCR+ group in the aSCR− group is upward.

The analysis of variance of repeated measures does not show significant effects of any main effect. Two interactions are significant, those obtained between SCR (anticipatory/post-election) and group (aSCR+/aSCR−) *F*_(2,24)_ = 9.348; *p* = 0.005, and between SCR (anticipatory/post-election) and blocks [*F*_(4,24)_ = 3.065; *p* = 0.036]. In the first interaction, we observe that there are no significant differences between anticipatory and post-election activation in the aSCR− group, while in the aSRC+ group if there are significant differences, anticipatory activation is slightly higher (*p* = 0.004). As for the relationship between the activation (anticipatory/post-election) along the blocks we obtain slightly lower levels in the post-election activation and in both cases (anticipatory and post-election activation) there is a slight downward trend.

**FIGURE 5:**
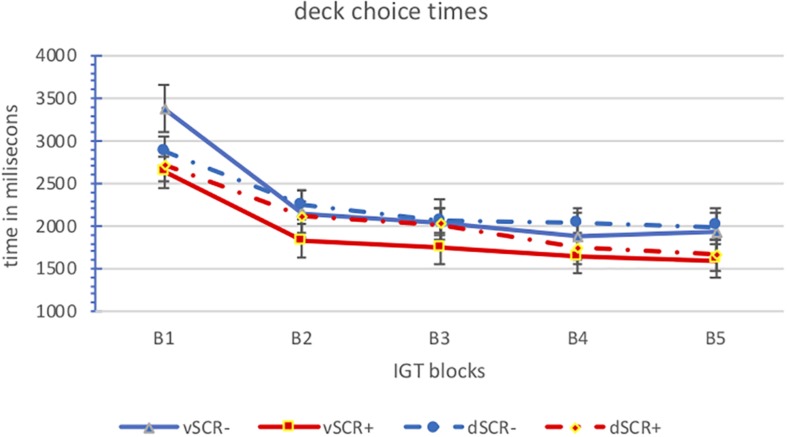
Means of the deck choice times by block for the positive (aSCR+) and negative (aSCR–) anticipatory skin conductance response groups for advantageous and disadvantageous deck (error bars represent standard error of the mean).

Finally, in relation to response times, we have analyzed the deck choice times by block for the positive (aSCR+) and negative (aSCR−) anticipatory skin conductance response groups for advantageous and disadvantageous deck (Figure 5 and Table 4). We can observe, both groups present descending election times, especially between the first and the second block. The aSRC+ group has the lowest response times in the advantageous elections.

**TABLE 4:** Mean and S.D. of deck choice times for advantageous and disadvantageous decks by blocks.

	**Advantageous (C+D)**				**Advantageous (A+B)**			
	**SCR−**		**SCR+**		**SCR−**		**SCR+**	
	**Mean**	**S.D.**	**Mean**	**S.D.**	**Mean**	**S.D.**	**Mean**	**S.D.**
B1	3368.642	1769.784	2632.038	1425.076	2876.044	930.469	2710.814	1010.941
B2	2152.657	889.514	1826.366	892.939	2246.659	996.518	2105.207	978.937
B3	2048.194	862.381	1741.432	717.125	2058.854	925.146	2022.747	916.060
B4	1875.548	708.558	1643.454	697.875	2033.633	878.071	1741.174	684.998
B5	1931.274	741.325	1593.584	604.072	1998.761	689.776	1667.924	645.841

The analysis of variance of repeated measures shows us only one main effect in the task blocks [*F*_(4.24)_ = 9.049; *p* = 0.000] confirming the observed downward trend, especially between the initial blocks (B1 and B2) and the rest.

## Discussion

The aim of this study was to determine whether healthy community-dwelling individuals would manifest a significantly distinct behavioral pattern in the IGT based on their SCRs prior to card selection, with the novelty that we did not establish a limited time interval between trials as a means to enhance the ecological validity of the task. Also, participants’ decisions were analyzed both with classical methods and a computational model.

Taken altogether, our results suggest that participants with higher anticipatory SCRs to the disadvantageous decks (aSCR+) perform better in the IGT than participants not showing such anticipatory responses (aSCR−). These results are similar to previous studies that apply limited time intervals between trials (Bechara et al., 1996, 1997, 2002; Bechara and Damasio, 2002; Carter and Pasqualini, 2004; Crone et al., 2004; Jenkinson et al., 2008; Starcke et al., 2009; Fonfría et al., 2015; Ottaviani and Vandone, 2015), suggesting that free time to deliberate responses does not change the decision-making process.

More important, it must be noted that the total GI of the aSCR− group, despite being healthy, is below the cut-off score (10) established by Bechara et al. (2001, 2002) and Bechara and Damasio (2002) for impaired decision making, and other studies have confirmed this criterion in different types of patients (Alameda et al., 2012; Cavedini et al., 2012). Although some studies have shown that different factors may explain this variability in healthy population, such as age (Denburg et al., 2009), level of education (Davis et al., 2008), gender (De Visser et al., 2010), certain personality characteristics (Glicksohn and Zilberman, 2010), anxiety (Fonfría et al., 2015), and the time or number of movements (Bull et al., 2015), our results reveal that special care must be taken regarding galvanic response profiles when selecting participants for control groups.

In addition, our results are consistent with the idea that the initial phase of the IGT is used to explore and learn how the task works (Damasio, 1994; Dunn et al., 2006). The aSCR+ and aSCR− groups start choosing differently only in the second block, indicating that participants with higher prior activation to the disadvantageous decks determine a better strategy in the decision process, resulting in a better performance in the IGT. The performance of the aSCR− group also improved across the task, with both groups behaving similarly in the last blocks, but at a different learning rate. Therefore, aSCR− participants may need more time to develop the appropriate strategy, as suggested by Bull et al. (2015) or Marin et al. (2019), they suggest that low SCR is related with hypoactivation of brain regions involved in fear learning. This being said, it would be interesting to see whether the group differences found would definitely disappear by extending the duration of the task.

Although the aSCR+ group prefers favorable decks, and participants with less activation to the disadvantageous decks select more unfavorable decks, when choices are analyzed in detail, we observe that the aSCR+ group prefers D, B, and C, ordered from highest to lowest preference. This means that these individuals could not establish B as a deck involving long-term losses (probably because they are focused on the frequency of the losses, which is one per 10 cards). This effect has been previously described in studies with non-clinical samples (e.g., Lin et al., 2007; Lee et al., 2014), and it underlines the importance of analyzing all four decks (ABCD), as argued by Steingroever et al. (2013). These decision-making problems are due to the inability to establish stimulus-reward relationships or to eradicate previously learnt responses (Maia and McClelland, 2004; Rolls, 2004). The choices of the disadvantageous decks offer gains at the beginning but losses in the long term. This shows that participants either did not adequately identify the characteristics of the decks (Fernie and Tunney, 2006; Lin et al., 2007, 2009) or they had problems eradicating their initial preference for the disadvantageous decks (A-B).

Not surprisingly, the aSCR− group prefers deck B, which may reveal hypersensitivity to reward (Bechara et al., 2002), but the next option is for deck D, which was less expected and would be consistent with punishment-avoidance interpretations (González et al., 2010). This is when PVL (maximum likelihood) parameters become useful. Analysis of the utility (α) parameter yields α-values near 0 in the aSCR+ group, showing that these participants are less sensitive to feedback outcomes than the aSCR− group. Similar results have been found in other studies (e.g., Alameda et al., 2012; Alameda-Bailén et al., 2014). However, data from the PVL simulations do not show significant group differences and, in both cases, the α-value denotes low sensitivity to feedback outcomes.

Regarding consistency (*c*), both groups score low on MLE and PVL-Decay, groups selects cards randomly, which is consistent with previous findings by Fridberg et al. (2010), although these authors focused on a different group comparison. The group differences are more evident in maximum likelihood than in simulation data but, in this case, the *c* values of both groups are similar.

Both groups are more sensitive to losses than to gains, although λ-values are higher with maximum likelihood than with PVL-Decay simulation. Finally, we observed the greatest difference between the maximum likelihood data and the simulation data in parameter A, which had lower values with no significant differences in the former and values close to 1 with significant group differences in the latter, and, although both groups grant more weight to recent outcomes.

## Conclusion

Summing-up, regardless of using unlimited inter-trial intervals, our results suggest that aSRCs to disadvantageous decks are indicating a somatic marker that guides the decision-making process toward more favorable choices, leading to a better score on the IGT. Furthermore, even in healthy participants, the decision-making process is qualitatively different in people who are well equipped to develop somatic markers (aSCR+) from people who are not (aSCR−). According to the PVL model parameters, decisions of participants who do not benefit from aSCRs to the disadvantageous decks are more random, and show low sensitivity to feedback outcomes. These findings are relevant for researchers using the IGT, as they highlight the potential effects of individual aSCR differences even in healthy control groups, and the failure to control for these differences, or at least to consider them in the data analysis, may lead to inaccurate behavioral results.

## Data Availability Statement

The raw data supporting the conclusions of this manuscript will be made available by the authors, without undue reservation, to any qualified researcher.

## Author Contributions

All authors contributed equally to the development of the manuscript, substantially to the design and planning, or analysis and interpretation of the data, significantly in drafting or critically reviewing the content, and participated in the approval of the final version of the manuscript.

## Conflict of Interest

The authors declare that the research was conducted in the absence of any commercial or financial relationships that could be construed as a potential conflict of interest.

## Supplementary Material

DATA SHEET S1 Database.
